# Surface Plasmon Resonance Sensor Based on Dual-Side Polished Microstructured Optical Fiber with Dual-Core

**DOI:** 10.3390/s20143911

**Published:** 2020-07-14

**Authors:** Haixia Han, Donglian Hou, Nannan Luan, Zhenxu Bai, Li Song, Jianfei Liu, Yongsheng Hu

**Affiliations:** 1 Tianjin Key Laboratory of Electronic Materials and Devices, School of Electronics and Information Engineering, Hebei University of Technology, Tianjin 300401, China; 201821902029@stu.hebut.edu.cn (H.H.); 201921902013@stu.hebut.edu.cn (D.H.); baizhenxu@hotmail.com (Z.B.); songli@hebut.edu.cn (L.S.); jfliu@hebut.edu.cn (J.L.); 2 State Key Laboratory of Luminescence and Applications, Changchun Institute of Optics, Fine Mechanics and Physics, Chinese Academy of Sciences, Changchun 130033, China; huyongsheng@ciomp.ac.cn

**Keywords:** microstructured optical fiber, optical fiber sensors, refractive index sensor, surface plasmon resonance

## Abstract

A surface plasmon resonance (SPR) sensor based on a dual-side polished microstructured optical fiber (MOF) with a dual core is proposed for a large analyte refractive index (RI; *n*_a_) detection range. Gold is used as a plasmonic material coated on the polished surface, and analytes can be directly contacted with the gold film. The special structure not only facilitates the fabrication of the sensor, but also can work in the *n*_a_ range of 1.42–1.46 when the background material RI is 1.45, which is beyond the reach of other traditional MOF-SPR sensors. The sensing performance of the sensor was investigated by the wavelength and amplitude interrogation methods. The detailed numerical results showed that the proposed sensor can work effectively in the *n*_a_ range of 1.35–1.47 and exhibits higher sensitivity in the *n*_a_ range of 1.42–1.43.

## 1. Introduction

Surface plasmon resonance (SPR) is a unique optical property, which refers to the resonance excitation of free electron oscillations at the interface between a metallic layer and a dielectric surface under a *p*-polarized light radiation, and is extremely sensitive to variations in the refractive index (RI) of the surrounding medium [1,2,3,4,5,6]. Therefore, SPR has been exploited with a wide range of sensing applications in different fields, such as biology, chemistry, and environmental medicine [1,2,3,4,5,6,7,8]. Initially, most SPR sensors were based on the Kretschmann–Raether prism geometry, but prism-based SPR sensing devices have the disadvantages of large volume, limited mechanical reliability, high cost, unsuitability for distributed sensing, and mass production, which are difficult to adapt themselves to the development of optical communication technology [1,2,3,4,5,6,7,8,9,10,11,12,13,14].

To effectively overcome the limitations of prism-based SPR sensors, optical fiber-based SPR sensors have been developed, in which the fiber acts as a prism, coupling incident light with plasmons. Compared with prism-based devices, the optical fiber design is simpler and more flexible, which can reduce the size of the sensor to a large extent. In addition, the advantages of electromagnetic immunity, high degree of integration, mechanical stability, and in situ monitoring have made optical fiber design more and more attractive [1,2,3,4,5,6,7,8,9,10,11,15,16]. However, the phase-matching condition between the core mode and the surface plasmon polariton (SPP) mode of optical fiber-based SPR sensors is difficult to meet. In principle, phase matching happens, when their effective refractive index (*n*_eff_) values of the two modes are identical. For single-mode fibers, the *n*_eff_ of the SPP mode is usually close to that of the adjacent analyte. For example, the *n*_eff_ of the water is 1.33, and the *n*_eff_ of the core mode is close to that of the core material, which for most practical materials is higher than 1.45 [15,16,17]. Therefore, phase matching occurs only at higher frequencies, and high frequencies limit the penetration depth of the plasmon into the analyte, which reduces the sensitivity of the sensor.

To alleviate phase-matching problems of optical fiber-based SPR sensors, microstructured optical fiber (MOF)-based SPR sensors have been widely studied owing to their design flexibility [9,12,13,14,15,16,17,18,19,20,21,22,23,24,25,26,27,28,29,30,31,32,33,34,35,36,37,38,39,40,41,42,43,44,45,46]. By changing the size, shape, and arrangement of air holes along the propagation direction, the *n*_eff_ can be tuned to the anticipated values and thus can solve the phase-matching problem between the core mode and the SPP mode [9,15,16,17,24,25]. In addition, due to their unique structural characteristics and novel optical properties, MOF-based SPR sensors can significantly improve sensing performance. However, there exists an undetectable range of the RI detection in MOF-based SPR sensors, which limits their application in biological and chemical sensing fields. For instance, when the background material RI of an MOF is 1.45, to satisfy the total reflection condition, the upper detection limit of the analyte RI (*n*_a_) is usually lower than 1.42 for low-RI MOF-SPR sensors [9,15,16,17,20,24,25,29], and the lower detection limit of *n*_a_ is usually higher than 1.45 for high-RI MOF-SPR sensors [30,31,32]. In addition, the size of air holes in these sensors is in the order of micrometers, which makes them difficult to be coated with metals or filled with analytes in actual manufacturing [4,6,9,12,15,16,19,24,25].

In this paper, to overcome the above problems, we propose an SPR sensor based on a dual-side polished MOF with a dual core. The dual-side polished structure can be coated with gold films and then directly contact with analytes, which helps to reduce the manufacturing difficulty of the sensor. The dual core can reduce the impact of total reflection conditions on the optical fiber, so that the sensor can work at a wide range of *n*_a_, especially at 1.42–1.46, in which range the other MOF-based SPR sensors cannot work [20,21,22,23,24,25,26].

## 2. Structure Design and Principle

**Figure 1:**
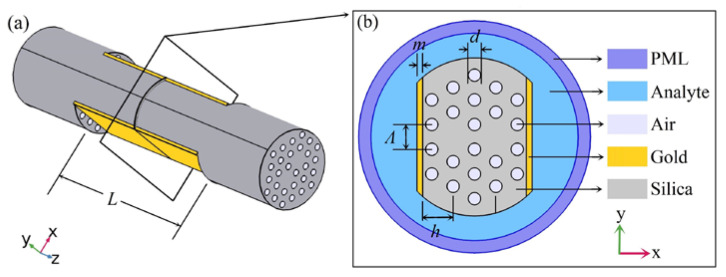
(**a**) Schematic diagram of the proposed dual-side polished microstructured optical fiber (MOF)-based surface plasmon resonance (SPR) sensor with a dual core; (**b**) cross-section of the proposed MOF-based SPR sensor.

The schematic diagram of the proposed SPR sensor based on a dual-side polished MOF with a dual core is depicted in Figure 1. This structure can be obtained by a wheel-polishing setup with a 3D mechanical platform, which can move along the X, Y, and Z directions [47]. By employing a light source and an optical spectrum analyzer to online monitor the transmission spectrum during the polishing process, the polishing position, length, and depth could be easy to set up and operate accurately via a computer program [46,47]. Gold was used as a plasmonic material to coat the polished surface, which is not as difficult as coating a gold film on the inner surface of small air holes. Here, the center-to-center distance between two adjacent air holes (Ʌ) was 3 μm, and the diameter of air holes (*d*) was 0.5Ʌ. The thickness of the gold film (*m*) was 40 nm, and the polishing depth from the fiber center to the polished surface (*h*) was 2.1Ʌ. The mode characteristics and the sensing performance of the proposed sensor were simulated through commercially available software COMSOL. A perfectly matched layer (PML) was added to the outer computational region, which was applied to absorb scattered light [9,10,15,16,18,19,25,26,27,28,29,31,36,37,38].

In this sensor, we used fused silica as a background material and set its RI at 1.45 [17,20,24,25,27,29,41] to detect a range of *n*_a_, which the other MOF-based SPR sensors cannot realize. The RI of the air was set to 1. To achieve the dielectric constant of gold (ε(*ω*)), we used the Drude–Lorentz model, of which the equation can be written as follows [48]:

(1) $$\varepsilon \left(\omega \right)=\varepsilon_{\infty }-\frac{\omega_{D}^{2}}{\omega \left(\omega +j\gamma_{D}\right)}+\frac{\Delta \varepsilon \cdot \Omega_{L}^{2}}{\left(\omega^{2}-\Omega_{L}^{2}\right)+j\Gamma_{L}\omega }$$

where ε_∞_ is the permittivity at high frequencies, *ω* can be interpreted as the angular frequency, *ω_D_* and *γ_D_* indicate the plasma frequency and the damping frequency, respectively, Δε is the weighting factor, and Γ*_L_* and Ω*_L_* are the spectral width and the oscillator strength of the Lorentz oscillators, respectively [10,34,36,48].

## 3. Simulation Results and Discussion

### 3.1. Coupling Properties

**Figure 2:**
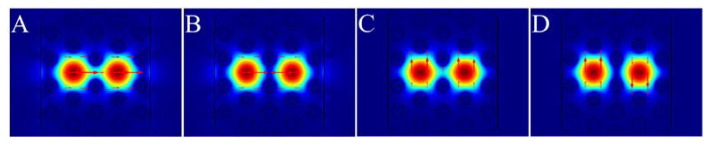
Electric field distributions of four fundamental supermodes at 1100 nm for the analyte refractive index (*n*_a_) of 1.44: (**A**) *x*-even mode; (**B**) *x*-odd mode; (**C**) *y*-even mode; (**D**) *y*-odd mode (the arrows represent the direction of the electric field).

Like other dual-core MOFs [37,38,39], the proposed sensor can support four supermodes in fundamental modes. Figure 2 shows the electric field distributions of the four supermodes at 1100 nm for *n*_a_ =1.44. Insets A and B of Figure 2 represent the even mode and the odd mode in the *x* polarized direction (*x*-even mode and *x*-odd mode), respectively. Insets C and D of Figure 2 represent the even mode and the odd mode in the *y* polarized direction (*y*-even mode and *y*-odd mode), respectively. Here, we only investigated the coupling properties of the *x*-polarized core mode, because the *y*-polarized core mode with the electric field was parallel to the gold film surface and was not easily coupled with SPP modes [39,40,41].

**Figure 3:**
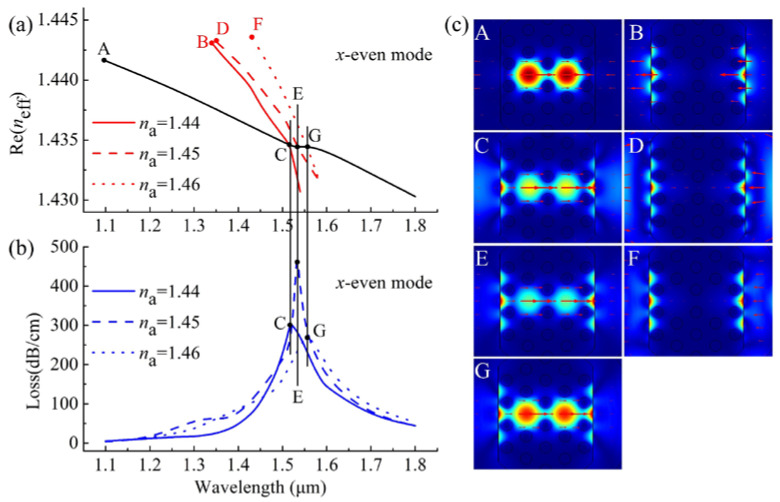
(**a**) The real parts of the effective refractive index (Re (*n*_eff_)) curves of the *x*-even core modes (black lines) and the *x*-even SPP modes at *n*_a_ = 1.44, 1.45, and 1.46 represented by the red solid, dashed, and dotted lines, respectively; (**b**) the loss spectra of the *x*-even core modes at *n*_a_ = 1.44, 1.45, and 1.46 represented by the blue solid, dashed, and dotted lines, respectively; (**c**) electric field distributions of the *x*-even core mode **A** at 1100 nm, *x*-even SPP mode **B** at 1340 nm, and *x*-even core mode **C** at 1518 nm for *n*_a_ = 1.44, electric field distributions of *x*-even SPP mode **D** at 1350 nm and *x*-even core mode **E** at 1533 nm for *n*_a_ = 1.45, and electric field distributions of *x*-even SPP mode **F** at 1430 nm and *x*-even core mode **G** at 1556 nm for *n*_a_ = 1.46.

In theory, when the real parts of *n*_eff_ (Re(*n*_eff_)) of the core mode and the SPP mode are equal, the phase-matching condition between them are satisfied. Then, the two modes will be coupled with each other, and the maximum energy transfer from the core mode to the SPP mode can be achieved [5,6,7,8,10,11,12,13,14,15,16,17,18,19,20,21,22,23,24,25,26,37]. Figure 3 shows the Re(*n*_eff_) curves of the *x*-even core modes and *x*-even SPP modes, the loss spectra of the *x*-even core modes, and the electric field distributions of the relevant modes for *n*_a_ = 1.44, 1.45, and 1.46. The black solid line represents the Re(*n*_eff_) of the *x*-even core mode, while the red solid, dashed, and dotted lines represent the Re(*n*_eff_) of the *x*-even SPP modes at *n*_a_ = 1.44, 1.45, and 1.46, respectively, as shown in Figure 3a. The blue solid, dashed, and dotted lines stand for the losses of the *x*-even core modes at *n*_a_ = 1.44, 1.45, and 1.46, respectively (Figure 3b). Take *n*_a_ equal to 1.44 as an example. The *x*-even core mode (inset A of Figure 3c) and the *x*-even SPP mode (inset B of Figure 3c) were coupled with each other (inset C of Figure 3c) at a wavelength of 1518 nm (point C in Figure 3a,b). At this wavelength (also called resonance wavelength), a significant loss peak appeared (see the blue solid curve in Figure 3b), which indicated the maximum energy transfer from the *x*-even core mode to the *x*-even SPP mode. The insets D, E, F, and G of Figure 3c represent the electric field distributions at points D, E, F, and G, respectively (Figure 3a,b), and they can also show the energy transfer from the *x*-even core mode to the *x*-even SPP mode at *n*_a_ = 1.45 and 1.46, respectively. As shown in Figure 3, the values of resonance wavelengths were shifted from 1518 to 1533 nm and from 1533 to 1556 nm due to the variations of *n*_a_ from 1.44 to 1.45 and from 1.45 to 1.46, respectively. We can observe that a tiny change of *n*_a_ can lead to a significant shift of resonance wavelength. This capability can be utilized to detect the changes of the analyte RI [19,24,25,26,37,38].

**Figure 4:**
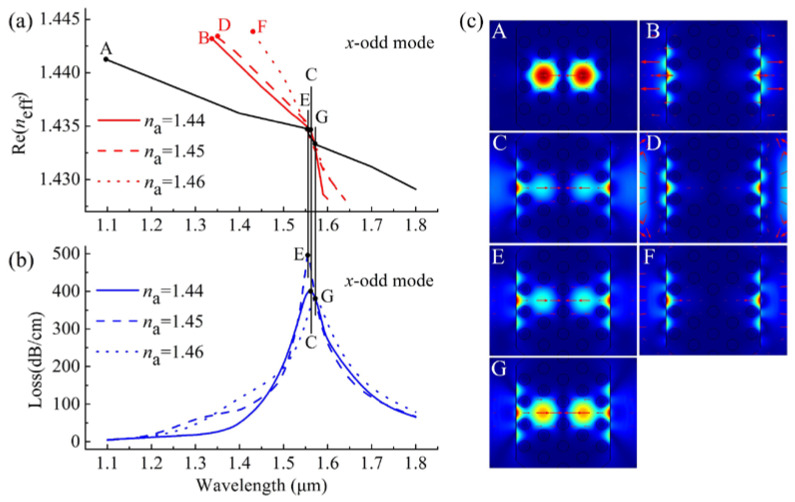
(**a**) The Re (*n*_eff_) curves of the *x*-odd core modes (black lines) and *x*-odd SPP modes at *n*_a_ = 1.44, 1.45,and 1.46 represented by the red solid, dashed, and dotted lines, respectively; (**b**) the loss spectra of the *x*-odd core modes at *n*_a_ = 1.44, 1.45, and 1.46 represented by the blue solid, dashed, and dotted lines, respectively; (**c**) electric field distributions of the *x*-odd core mode **A** at 1100 nm, the *x*-odd SPP mode **B** at 1340 nm, and the *x*-odd core mode **C** at 1561 nm for *n*_a_ = 1.44, electric field distributions of *x*-odd SPP mode **D** at 1350 nm and *x*-odd core mode **E** at 1556 nm for *n*_a_ = 1.45, and electric field distributions of *x*-odd SPP mode **F** at 1430 nm and *x*-odd core mode **G** at 1572 nm for *n*_a_ = 1.46.

When varying the *n*_a_ from 1.44 to 1.46, the Re(*n*_eff_) curves of the *x*-odd core modes (black solid lines) and the *x*-odd SPP modes (red solid, dashed, and dotted lines) and the loss spectra (blue solid, dashed, and dotted lines) of the *x*-odd core modes, as well as the electric field distributions of the relevant modes are shown in Figure 4. Similar to the coupling properties of the *x*-even core modes, the phase-matching conditions between the *x*-odd core mode and *x*-odd SPP modes were satisfied at the wavelength of 1518 nm (point C in Figure 4a,b) for *n*_a_ = 1.44, at the wavelength of 1533 nm (point E in Figure 4a,b) for *n*_a_ = 1.45, and at 1556 nm (point G in Figure 4a,b) for *n*_a_ = 1.46. At the resonance wavelengths, the corresponding electric field distributions are shown in insets C, E, and G of Figure 4c, when the *n*_a_ values were 1.44, 1.45, and 1.46, respectively. Compared with Figure 3a,b, we found that, unlike the resonance wavelength of the *x*-even core mode shifting to longer wavelengths with *n*_a_ increasing from 1.44 to 1.46, the resonance wavelength of the *x*-odd core mode moved to a shorter wavelength as *n*_a_ varied from 1.44 to 1.45, whereas the resonance wavelength moved towards longer wavelengths as *n*_a_ increased from 1.45 to 1.46. This unexpected peak behavior disturbs the regularity of the SPR sensor and therefore cannot be utilized to detect the changes of the analyte RI.

From Figure 3 and Figure 4, we can see that the resonance wavelengths of the *x*-even core modes move toward longer wavelengths with increasing *n*_a_, but the behavior of the resonance wavelengths of the *x*-odd core modes is not regular and cannot be used to detect the variations of the analyte RI. In the following discussion, we only consider the sensing performance of the *x*-even core modes.

### 3.2. Sensing Performance

The sensing performance of the sensor can be evaluated by wavelength sensitivity (wavelength interrogation) and amplitude sensitivity (amplitude interrogation) [5,10,15,18,26]. The wavelength sensitivity can be calculated from the following equation [5,10,11,12,13,14,15,17,18,19,20,26,37,38]:

(2) $$S(nm/RIU)=\frac{\Delta \lambda_{peak}}{\Delta n_{a}}$$

where Δ*λ_peak_* is the shift of the resonance wavelength and Δ*n*_a_ is the variation of *n*_a_. As shown by the blue solid and dashed lines in Figure 3b, we observed Δ*λ_peak_* of 15 nm when *n*_a_ was varied from 1.44 to 1.45. According to Equation (2), the corresponding wavelength sensitivity in terms of refractive index units (RIU) was 1500 nm/RIU.

**Figure 5:**
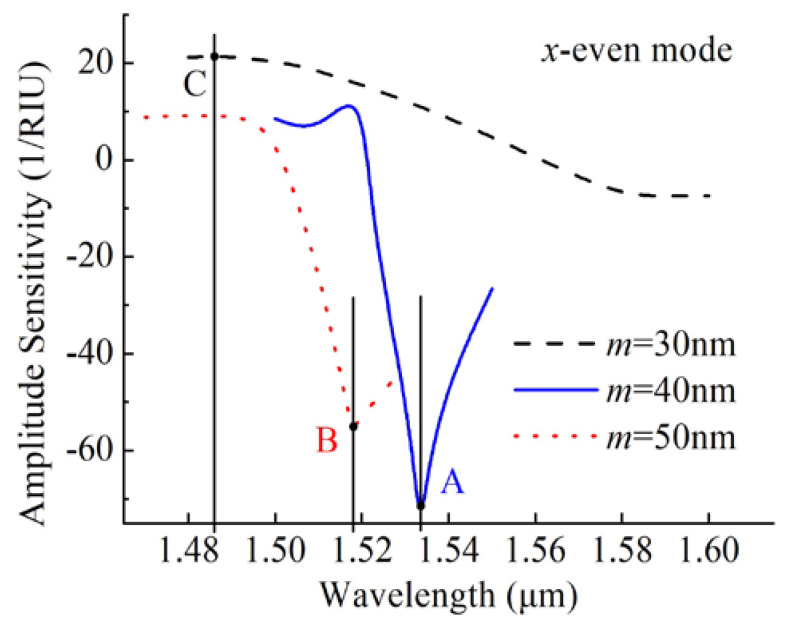
Amplitude sensitivities of *x*-even core modes for the gold film thicknesses of 30, 40, and 50 nm.

The amplitude sensitivity can be calculated at a particular wavelength. Assuming a reasonable length of the sensor was L = 1/α(*λ*, *n*_a_), the amplitude sensitivity was expressed as [5,10,17,18,26,29,38]:

(3) $$S(RIU^{-1})=-\frac{1}{\alpha (\lambda ,n_{a})}\frac{\partial \alpha \left(\lambda ,n_{a}\right)}{\partial n_{a}}$$

where α (*λ*, *n*_a_) is the overall loss for a particular wavelength, ∂α(*λ*, *n*_a_) is the difference between two adjacent loss spectra due to a small change in *n*_a_, and ∂*n*_a_ is the change of *n*_a_. According to Equation (3), we plotted the amplitude sensitivity curves in Figure 5. As is shown by the blue solid curve, the maximum amplitude sensitivity of the *x*-even core mode was 72.18 RIU^–1^ at 1534 nm for *m* = 40 nm.

### 3.3. Gold Film Thickness

**Figure 6:**
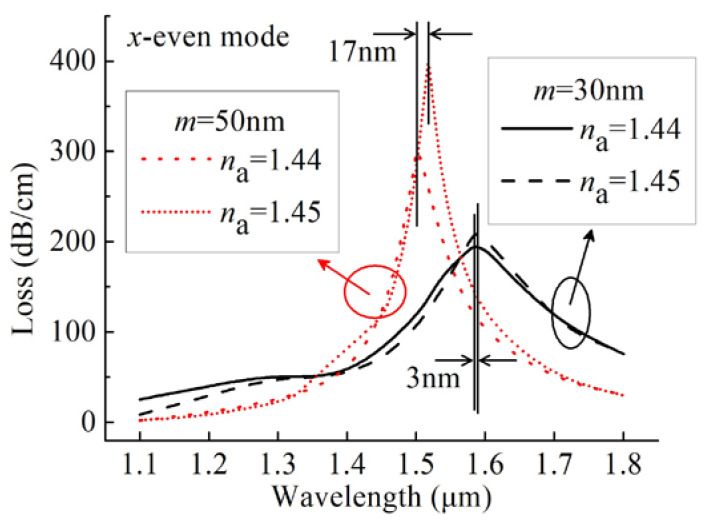
Loss spectra of the *x*-even core modes for the gold film thicknesses of 30 and 50 nm at *n*_a_ of 1.44 and 1.45.

The thickness of a gold film is the most important factor that affects the SPR spectra and thus the sensing performance [8,9,10,12,13,15,16,18,24,33,34,35,36]. Figure 6 shows the loss spectra of the *x*-even core modes by varying *m* for *n*_a_ = 1.44 and 1.45. Comparing Figure 3b and Figure 6, it can be evident that the resonance wavelength moved to a shorter wavelength by increasing *m* from 30 to 50 nm in the case of *n*_a_ = 1.44 and 1.45. According to Equation (2), the values of the wavelength sensitivities were 300 and 1700 nm nm/RIU for *m* of 30 and 50 nm, respectively. The peak losses and the wavelengths influenced by varying *m* also affected the amplitude sensitivities. Figure 5 shows the amplitude sensitivities of *x*-even core modes as *m* varied from 30 to 50 nm. It can be found that the maximum amplitude sensitivities of 21.35 and 55.3 RIU^–1^ were achieved at 1486 and 1518 nm for *m* of 30 and 50 nm, respectively.

To further evaluate the performance of the designed sensor, Table 1 shows the summary of several sensing parameters including the peak wavelength, the peak loss, the wavelength sensitivity, the maximum amplitude sensitivity, and the wavelength for the maximum amplitude sensitivity of different *m* at an *n*_a_ range of 1.35–1.47. By means of a detailed investigation of theses parameters, it was found that *m* can affect the peak wavelength and the peak loss and therefore affect the wavelength sensitivity, the maximum amplitude sensitivity, and the wavelength for the maximum amplitude sensitivity. It is worth noting that the trend of the wavelength for the maximum amplitude sensitivity was roughly the same as that of the peak wavelength, because according to Equation (3), the maximum amplitude sensitivity is related to the maximum ∂α (*λ*, *n*_a_), which generally occurs in the vicinity of the resonance peak. The change of *m* has a slight impact on the sensing performance in single-core MOF-SPR sensors [9,10,12,13,15,16,24,33], while it has a greater and more irregular impact on the sensing performance in the proposed dual-core MOF-SPR sensor, which indicates that this dual-core structure is more sensitive to the thicknesses of the gold film. Nevertheless, the designed dual-core MOF sensor can detect a larger RI range, and it exhibits higher wavelength and amplitude sensitivities at the *n*_a_ range of 1.41–1.43, even with other gold film thicknesses.

In general, the ability of detecting large RI ranges makes the designed sensor more competitive than D-shaped MOF-SPR sensors [17,21,22,23,32,33,34,38]. Compared with single-core dual-side polished MOF-SPR sensors [18,42], the designed dual-core structure can detect *n*_a_ higher than the RI of the background material of the MOF. Although other structures such as inner-coated or grooved MOF-SPR sensors can detect a wide RI range [19,39,43,44,45], the designed dual-side polished structure has advantages that it can be readily coated with the gold films and has outside sensing channels, making it ideal for use as a real-time sensor.

## 4. Conclusions

We have proposed and numerically investigated an SPR sensor based on a dual-side polished MOF with a dual core to realize a large range of *n*_a_ detection. The gold and the analyte layers were placed outside the MOF structure, which can be expected to simplify the manufacturing process. The coupling characteristics, sensing performance, and the influence of the gold film thickness of the sensor were investigated by the finite element method in the wavelength and amplitude interrogation. Since the peak behavior of the *x*-odd mode disturbed the regularity of the SPR sensor, the *x*-even mode was determined to analyze the sensing performance. The simulation results showed that the sensor could detect a large *n*_a_ range covering from 1.35 to 1.47 and had higher wavelength sensitivity and amplitude sensitivity in the *n*_a_ range of 1.42–1.43 when the background material RI was 1.45. The proposed sensor can overcome the defect that other traditional MOF-SPR sensors cannot work in the *n*_a_ range of 1.42–1.46, which makes it exhibit great potential in biological and chemical sensing fields.

## Figures and Tables

**Table 1:** Comparison of the sensing parameters of the *x*-even core modes at the *n*_a_ range of 1.35–1.47 for *m* values of 30, 40, and 50 nm, respectively.

		Peak Wavelength (nm)			Peak Loss (dB/m)			Wavelength Sensitivities (nm/RIU)			Maximum Amplitude Sensitivities (RIU–1)			Wavelength for the Maximum Amplitude Sensitivity (nm)		
	*m*	30 nm	40 nm	50 nm	30 nm	40 nm	50 nm	30 nm	40 nm	50 nm	30 nm	40 nm	50 nm	30 nm	40 nm	50 nm
*n*_a_																
1.35		1107	1124	967	400.46	303.1	332.45	900	600	300	16.29	8.27	13.36	1132	1141	985
1.36		1116	1130	970	420.74	319.45	337.37	1000	700	400	19.41	10.69	17.35	1141	1146	989
1.37		1126	1137	974	449.8	343.09	346.69	1200	800	600	23.97	14.62	24.16	1152	1154	994
1.38		1138	1145	980	493.53	379.69	365.6	1400	1100	800	31.11	21.73	37.8	1165	1164	1001
1.39		1152	1156	988	563.82	442.55	407.66	1800	1300	1300	43.47	36.78	73.6	1182	1178	1012
1.40		1170	1169	1001	688.61	568.95	523.56	2200	2200	12000	67.89	77.88	100.9	1202	1198	1127
1.41		1192	1191	1121	946.43	913.32	748.38	3100	3100	4900	126.83	270.71	852.92	1230	1231	1171
1.42		1223	1225	1170	1634.6	2674.4	5094.5	17500	17500	20700	407.64	1479.03	1411.37	1408	1340	1377
1.43		1398	1340	1377	6944.1	18113	29443	18700	18700	12400	1262.38	1347.09	390	1591	1519	1503
1.44		1585	1518	1501	19434	30453	30473	300	300	1700	21.35	72.18	55.39	1486	1534	1518
1.45		1588	1533	1518	20922	48818	40144	5600	5600	600	42.18	52.2	54.06	1556	1533	1524
1.46		1644	1556	1524	16769	26788	54836	2600	2600	300	71.72	11.69	4.3	1482	1541	1521
1.47		1670	1565	1527	16005	24814	47015									
